# Peripheral blood leukocytes express differential chemokine receptor profiles in irritable bowel syndrome, Crohn’s disease and ulcerative colitis

**DOI:** 10.1186/1479-5876-10-S3-P13

**Published:** 2012-11-28

**Authors:** Ludvig Linton, Emma Lindh, Hans Glise, Michael Eberhardson, Ola Winqvist

**Affiliations:** 1 Dept. of Medicine, Translational Immunology Unit, Karolinska Institutet, Stockholm, Sweden; 2 Dept. of Gastroenterology and Hepatology, Karolinska University Hospital Solna, Stockholm, Sweden

## Background

Inflammatory bowel disease (IBD) is a collective term, generally referring to ulcerative colitis (UC) and Crohn’s disease (CD). Distinguishing between the sub-diagnoses of IBD as well as differential diagnoses such as irritable bowel syndrome (IBS) poses a challenge to the gastroenterologist because of the large overlap in clinical features. Making a correct diagnosis is important because treatment alternatives differ greatly between the two entities. Still, many patients are mis-diagnosed every year, and thus, there is need for developing improved diagnostic methods. In this study, we have used flow cytometry to investigate the expression of 20 chemokine receptors on the CD3+, CD14+ and CD19+ peripheral blood leukocyte populations in patients with UC, CD and IBS.

## Materials and methods

44 subjects were included in this study (HC=20; UC=4; CD=11; IBS=9). Leukocytes were isolated from heparinized whole blood by red blood cell lysis using NH_4_Cl buffer. Cells were Fc-blocked and subsequently stained with fluorochrome-conjugated antibodies against 20 chemokine receptors as well as leukocyte subset markers. Expression was normalized against fluorochrome-matched isotype controls for all parameters. Samples were analyzed on a FACSCanto flow cytometer (BD Biosystems). Statistical analyses were carried out using two-tailed independent t-test in Statistica 10 software (Statsoft).

## Results

9 parameters were found to be differentially expressed between the IBS and IBD groups. Here, CXCR4 on CD19+ B cells provided the largest group separation (p<0.001). Between UC and CD, 12 parameters were able to distinguish the diagnoses with CXCR5 on CD14+ monocytes accounting for the largest difference in expression.

## Conclusion

Our results show that chemokine receptor expression profiles on peripheral blood leukocyte subsets differ significantly between patients diagnosed with UC, CD and IBS. Larger studies are needed to evaluate the benefit of using flow cytometry analyses of chemokine receptors as a diagnostic complement in IBD/IBS.

Irritable bowel syndrome (IBS) is a symtom-based diagnosis characterized by alternating diarrhoea and constipation, abdominal pain and great discomfort. The causative mechanisms of IBS are unknown, but it has been suggested that IBS is in fact early-stage inflammatory bowel disease (IBD). Due to the large overlap in clinical features, distinguishing IBD from IBS poses a challenge for the gastroenterologist.

Around one in five Australians experiences the unpleasant symptoms of irritable bowel syndrome (IBS) at some time. These include abdominal pain, mucus in the stools, and alternating diarrhoea and constipation. Other terms for irritable bowel syndrome include ‘spastic colon’ and ‘irritable colon’. It seems that people with IBS have sensitive bowels that are easily ‘upset’. More women than men are prone to IBS, and symptoms tend to first occur in early adulthood.

The cause is unknown, but environmental factors such as changes of routine, emotional stress, infection and diet can trigger an attack. Research has shown that the neurotransmitter serotonin may be important in the symptoms of IBS, by altering the function of nerve cells in the bowel and causing changes in pain sensation and bowel function.

